# Study on the correlation between mineral bone metabolism and CRP in patients with SHPT during perioperative period

**DOI:** 10.1002/iid3.828

**Published:** 2023-04-12

**Authors:** Lei Yan, Qiuyue Xiong, Qin Xu, Pengru Ren, Tiantian Li, Huixia Cao, Fengmin Shao

**Affiliations:** ^1^ Department of Nephrology, Zhengzhou University People's Hospital, Henan Provincial People's Hospital, Henan Provincial Key Laboratory of Kidney Disease and Immunology Henan Provincial Clinical Research Center for Kidney Disease Zhengzhou China

**Keywords:** chronic kidney disease, inflammation, mineral bone metabolism disorders, parathyroidectomy, secondary hyperparathyroidism

## Abstract

**Objective:**

This study mainly observes changes in perioperative mineral bone metabolism‐related indicators and inflammatory factors in patients with secondary hyperparathyroidism (SHPT), and analyzed the correlation between mineral bone metabolism‐related indicators and inflammatory factors.

**Methods:**

Clinical data were collected. The study detects mineral bone metabolism‐related indicators and inflammatory factor of perioperative patients with SHPT before and 4 days after operation. The production of high‐sensitivity c‐reactive protein (hs‐CRP) in human hepatocytes cells (LO2 cells) stimulated by different concentrations of parathyroid hormone‐associated protein was detected by enzyme‐linked immunosorbent assay, reverse‐transcription polymerase chain reaction (RT‐PCR), and western blot.

**Results:**

The levels of mineral bone metabolism‐related indicators and hs‐CRP in SHPT group were significantly higher than those of control group. After operation, serum calcium, serum phosphorus, iPTH, FGF‐23 decreased, and the level of osteoblast active biomarkers increased, while the level of osteoclast active biomarkers decreased. The levels of hs‐CRP decreased significantly after operation. With the increase of PTHrP concentration, hs‐CRP level in supernatant of LO2 cells decreased first and then increased. RT‐PCR and western blot shows the same trend.

**Conclusion:**

Parathyroidectomy can significantly improve bone resorption and inflammation in SHPT patients. We speculate that there may be an optimal range of PTH concentrations to minimize inflammation in the body.

## Graphical abstract

Parathyroidectomy can significantly improve bone resorption and inflammation in secondary hyperparathyroidism (SHPT) patients. We speculate that there may be an optimal range of parathyroid hormone (PTH) concentrations to minimize inflammation in the body.

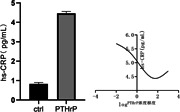

## 1 INTRODUCTION

Chronic kidney disease (CKD) has become a public health problem in the world, and the incidence of CKD is increasing year by year. The greatest number of adults living with CKD were in China (up to 159.8 million) and India (up to 140.2 million), collectively having 69.1% of the total number of adults with CKD in the region.^1^ As one of the most common complications of CKD, mineral bone metabolism abnormality increases fracture risk, greater morbidity, and mortality.^2^

Abnormal mineral bone metabolism begins in early stages of CKD and finds its maximum expression in stages 4 and 5.^3^ Secondary hyperparathyroidism (SHPT) is the main component of mineral bone metabolism disorders in CKD, and is closely associated with cardiovascular disease, fracture, and high mortality risk in end‐stage renal disease (ESRD).^4^

Parathyroid hormone (PTH) is synthesized and secreted by the parathyroid gland. Under normal circumstances, calcium ions in extracellular fluid bind to calcium‐sensitive receptor (CASR) in the plasma membrane of parathyroid cells to reduce the secretion of PTH.^5^ PTH reflects parathyroid function and is involved in mineral metabolism. SHPT and hyperphosphataemia are independently associated with CKD progression and the incidence of cardiovascular event in CKD patients.^6^ The abnormality of mineral bone metabolism in SHPT patients is manifested in the abnormality of bone formation markers and bone absorption markers, the typical representative of the former is type I collagen amino‐terminal propeptide (PINP) and bone alkaline phosphatase (BAP), while the latter mainly consists of type I collagen C‐Terminal peptide (CTX‐I) and tartrate‐resistant acid phosphatase (TRAP)‐5b. Clinically, for patients with advanced and severe refractory SHPT, drug therapy is usually ineffective. Parathyroidectomy (PTX) is a effective therapy that can reduce the level of PTH, calcium, phosphorus and relieve bone pain and pruritus.^7^

Microinflammatory state is a noninfectious, persistent, chronic immune inflammatory response centered on the release of circulating proinflammatory cytokines. Chronic inflammation, oxidative stress, and mitochondrial dysfunction play key roles in the progression and pathophysiology of CKD.^8^ Biomarkers of inflammation (IL, CRP) is independently associated with incident atherosclerotic vascular disease events and death in patients with CKD.^9^ CRP is one of the most basic inflammatory markers and is associated with many chronic diseases. The IFLS study in Asian populations showed that the elevated level of high‐sensitivity c‐reactive protein (hs‐CRP) is significantly associated with the risk of diabetes, heart disease, hypertension, and kidney disease.^10^ SHPT is a common complication in patients with end‐stage renal disease. SHPT is characterized by elevated concentrations of fibroblast growth factor 23 and serum parathyroid hormone (PTH), decreased concentrations of 1,25 (OH)2 vitamin D, and abnormal serum phosphorus (P) and calcium (Ca) concentrations.^11^ In kidney disease, overexpression of CRP can cause kidney cell damage and renal fibrosis.^12^ Serum CRP levels are significantly elevated in patients with CKD,^13^ and gradually increasing CRP levels over time are associated with increased risk of hospitalization in patients with CKD,^14^ and hs‐CRP is associated with risk of cardiovascular events and mortality in patients with CKD.^15^ In addition, studies have shown that FGF‐23 levels are positively correlated with hs‐CRP,^16^ Serum 25‐hydroxyvitamin D is negatively correlated with CRP in patients with vitamin D deficiency,^17^ and hs‐CRP levels in the severe SHPT group are higher than those in the mild SHPT group.^18^ In summary, CKD, the increase of FGF‐23, and vitamin D deficiency are all associated with the occurrence of SHPT, and they are all associated with CRP. Therefore, it is reasonable to believe that there is inflammation in the body of patients with SHPT, and the risk of disease and poor prognosis may be related to the increased burden of CRP.

Current studies have mainly analyzed the relationship between abnormal mineral bone metabolism indexes and microinflammatory state in patients with different stages of CKD or patients who have entered dialysis treatment stage, but unfortunately the results are inconsistent, and there are no reports regarding perioperative SHPT patients as research objects. Most studies have suggested that PTH is correlated with the inflammatory state and oxidative stress of the body.^19^, ^20^ However, in a study involving 155 patients with regular hemodialysis (HD), PTH showed no correlation with oxidative stress parameters and inflammatory factors of the subjects.^21^ Therefore, we want to study the relationship between PTH and inflammation.

This study mainly observes the correlation between the perioperative mineral bone metabolism related indicators and inflammatory factors in patients with refractory SHPT, and further verify whether mineral bone metabolism‐related indicators involved in the regulation of inflammatory cytokines by cell in vitro experiments, to provide the theory basis for the diagnosis and treatment of CKD‐mineral and bone disorder (CKD‐MBD), and to improve the patient survival rate and quality of life.

## 2 MATERIALS AND METHODS

### 2.1 Clinical data collection and testing

A total of 47 patients from Henan Provincial People's Hospital who needed to be hospitalized for parathyroid resection due to refractory secondary hyperparathyroidism from 2018 to 2019 were selected, and 20 healthy people from the same period were selected as the control group. Clinical data of SHPT group were collected, including gender, age, body mass index, hemoglobin, albumin, total cholesterol, triglyceride, hemopoietin (EPO), ferritin, serum calcium, serum phosphorus, and serum iPTH. Fasting blood was collected preoperatively (the first day after admission) and 4 days after surgery, and centrifuged at room temperature (3000 rpm, 10 min) within 30 min. Serum and plasma were collected and divided into 200 μL centrifuge tubes, marked, and placed at −80°C for later use. Vein blood samples, age, height, weight, and other data of the control group were collected.

### 2.2 Perioperative management

(1) Under regular dialysis, HD patients received heparin‐free dialysis once within 24 h before surgery; peritoneal dialysis (PD) patients were given continuous nonbedridden abdominal dialysis 24 h before surgery.
(2) Before skin incision after general anesthesia and immediately after surgery (after all parathyroidectomy), venous blood of the patients was collected 10 min after surgery and 20 min after surgery, and immediately sent to the isotope room for serum iPTH detection. The criteria for successful surgery were as follows: Serum iPTH level decreased by at least 82.9% 10 min after surgery or 88.9% 20 min after surgery compared with before surgery,^22^ which assisted in the evaluation of surgical efficacy.

### 2.3 Determination of mineral bone metabolism‐related indexes and inflammatory markers (enzyme‐linked immunosorbent assay)

Reference range of relevant indicators

The reference values of BAP (IDS) were 3.27–19.27 μg/L. The reference values of PINP (Guangzhou Feikang Biotechnology Co., Ltd.) were 30.40–65.20 μg/L; the reference value of CTX‐I (Guangzhou Feikang Biotechnology Co., Ltd.) was 0.10–0.71 μg/L. The reference value of TRAP‐5b (Guangzhou Feikang Biotechnology Co., Ltd.) is 1.18–4.02 U/L. The reference value of iPTH is 12–88 pg/mL; the reference value of FGF‐23 (Immutopics) is 21–82 RU/mL; the reference value of CRP is 0–10 mg/L.

### 2.4 Effects of parathyroid hormone‐associated protein (PTHrP) stimulation on the level of inflammatory factor hs‐CRP in LO2 cells in vitro experiments

It observes the effect of different concentrations of PTHrP (PeproTech) on the expression of inflammatory factors in LO2 cells. Well‐grown LO2 cells were cultured with culture medium (95% Dulbecco's modified Eagle's medium [Gibco] and 5% fetal bovine serum [BI]) to 60%–70%, then the medium was updated and 20 μL of PTHrP at different concentrations (10^−2^, 10^−1^, 1, 10, and 10^2^ ng/mL) was added. The blank control group was set to add the same amount of culture medium. Different groups were placed simultaneously in incubator (containing 5% CO_2_) at 37°C for 24 h, and then the supernatant and cells were collected. The levels of inflammatory factor hs‐CRP were determined by enzyme‐linked immunosorbent assay (Elabscience) and reverse‐transcription polymerase chain reaction (RT‐PCR) (forward, CGCAGTCCCTCCACTTTCTC; reverse, TCACAGCCCCACAAGGT‐TC), western blot (CRP,25kd; Abcam).

### 2.5 Statistic analysis

Statistical software SPSS23.0 was used to conduct statistical analysis on the data. Shapiro–Wilk test was used to identify whether variables are normally distributed. The measurement data conforming to normal distribution was expressed as mean ± standard deviation (*X* ± *S*), and the data conforming to nonnormal distribution was expressed as quaternary (*M* (Q1, Q3)). The independent sample *t* test was used for the comparison between the two groups conforming to the normal distribution, while the rank sum test was used for the comparison between the two groups conforming to the nonnormal distribution. Qualitative data were tested by *χ* ^2^ test. Spearman or Pearson analysis was used for correlation analysis, and *p* < .05 was considered statistically significant.

## 3 RESULTS

### 3.1 The baseline results

#### 3.1.1 Baseline data of SHPT group and control group

There was no significant difference in age and sex composition between SHPT group and control group. Body mass index in SHPT group was significantly lower than that in control group (*p* < .001). Forty‐seven patients in the SHPT group were dialysis aged (7.11 ± 2.84) years, including 43 patients with HD and four patients with PD. The levels of hemoglobin and serum albumin were generally low. The levels of total cholesterol and triglyceride were within the normal reference range. Erythropoietin was high in the normal range; Ferritin levels were above the normal range; the levels of alkaline phosphatase, serum phosphorus, serum calcium, iPTH, FGF‐23, PINP, BAP, CTX‐I, and TRAP‐5b were significantly increased, as detailed in Table 1.

**Table 1:** Baseline data of SHPT group and control group/normal reference range.

	SHPT group	Control/normal reference range
Age, y	47.44 ± 9.89	46.10 ± 5.89
Sex ratio	1.35	1.5
BMI, kg/m^2^	20.14 ± 1.52	23.91 ± 2.03
Hb, g/L	100.92 ± 20.61	130–175/120–165
ALB, g/L	36.93 ± 5.01	40–55
EPO, mIU/mL	11.79 (7.25, 53.62)	2.59–18.50
FERRITIN, ng/mL	516.30 (128.33, 1345.75)	23.90–336.20
CHOL, mmol/L	4.09 ± 0.77	2.33–5.17
TG, mmol/L	1.42 (1.06, 2.00)	0.00–1.70
ALP, U/L	752.50 (402.25, 1268.00)	45.00–125.00
P, mmol/L	2.29 ± 0.59	0.85–1.51
Ca, mmol/L	2.40 ± 0.27	2.11–2.52
iPTH, pg/mL	2503.80 (1925.10, 3088.00)	12.00–88.00
FGF‐23, RU/mL	8756.40 (5390.00, 30226.29)	21.00–82.00
PINP, μg/L	4707.91 ± 2448.35	30.40–65.20
BAP, μg/L	194.85 (88.65, 315.72)	3.27–19.27
CTX‐I, μg/L	1.32 (1.14, 1.58)	0.10–0.71
TRAP‐5b, U/L	15.15 (6.54, 24.05)	1.18–4.02
hs‐CRP, mg/L	13.14 (9.71, 15.62)	0–10

*Note*: Values are expressed as mean ± standard deviation, median (interquartile interval). The levels of alkaline phosphatase, serum phosphorus, serum calcium, iPTH, FGF‐23, PINP, BAP, CTX‐I, and TRAP‐5b were significantly increased.

Abbreviations: ALB, serum albumin; ALP, alkaline phosphatase; BAP, bone alkaline phosphatase; Ca, serum calcium; CHOL, total cholesterol; CTX‐I, type I collagen C‐terminal peptide; EPO, erythropoietin, FERRITIN; Hb, hemoglobin; P, serum phosphorus; PINP, type I collagen amino‐terminal propeptide; TG, triglyceride; TRAP, tartrate‐resistant acid phosphatase.

#### 3.1.2 Correlation analysis of preoperative‐related indexes in SHPT patients

Correlation analysis of iPTH, FGF‐23, and clinic data in SHPT patients. Preoperative serum iPTH of SHPT patients was positively correlated with dialysis age (*r* = .558, *p* < .001) and ALP (*r* = .471, *p* = .001). Serum iPTH was also positively correlated with PINP (*r* = .374, *p* = .016), BAP (*r* = .527, *p* = .002), CTX‐I (*r* = .400, *p* = .010), FGF‐23 (*r* = .644, *p* = .001), hs‐CRP (*r* = .801, *p* < .001). FGF‐23 was associated with dialysis age (*r* = .434, *p* = .034), P (*r* = .453, *p* = .029), hs‐CRP (*r* = .579, *p* = .004), See Table 2.

**Table 2:** Correlation analysis of preoperative serum iPTH, FGF‐23, and clinic data in SHPT patients.

Dependent variable	Independent variable	*r* Value	*p* Value
iPTH	BMI	.330	.038
	Dialysis age	.558	<.001
	Hb	−.013	.938
	ALB	.153	.345
	FERRITIN	−.147	.378
	CHOL	−.013	.941
	TG	.058	.738
	EPO	−.041	.832
	ALP	.471	.001
	P	.137	.394
	Ca	−.077	.633
	FGF‐23	.644	.001
	PINP	.374	.016
	BAP	.527	.002
	CTX‐I	.400	.010
	TRAP‐5b	.301	.066
	hs‐CRP	.801	<.001
FGF‐23	BMI	.402	.057
	Dialysis age	.434	.034
	Hb	−.373	.079
	ALB	.107	.628
	EPO	.465	.070
	FERRITIN	−.411	.052
	CHOL	.129	.599
	TG	.147	.547
	ALP	.214	.316
	P	.453	.029
	Ca	−.122	.590
	PINP	.208	.340
	BAP	.119	.597
	CTX‐I	.265	.222
	TRAP‐5b	.276	.214
	hs‐CRP	.579	.004

*Note*: Preoperative serum iPTH of SHPT patients was positively correlated with dialysis age (*r* = .558, *p* < .001) and ALP (*r* = .471, *p* = .001). Serum iPTH was also positively correlated with PINP (*r* = .374, *p* = .016), BAP (*r* = .527, *p* = .002), CTX‐I (*r* = .400, *p* = .010), FGF‐23 (*r* = .644, *p* = .001), hs‐CRP (*r* = .801, *p* < .001). FGF‐23 was associated with dialysis age (*r* = .434, *p* = .034), P (*r* = .453, *p* = .029), hs‐CRP (*r* = .579, *p* = .004).

Abbreviations: ALB, serum albumin; ALP, alkaline phosphatase; BAP, bone alkaline phosphatase; Ca, serum calcium; CHOL, total cholesterol; CTX‐I, type I collagen C‐terminal peptide; EPO, erythropoietin, FERRITIN; Hb, hemoglobin; P, serum phosphorus; PINP, type I collagen amino‐terminal propeptide; TG, triglyceride; TRAP, tartrate‐resistant acid phosphatase.

**Figure 1:**
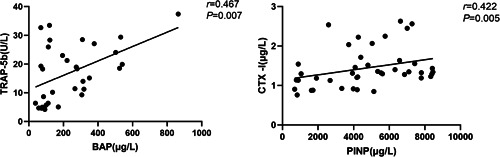
Correlation analysis of preoperative bone metabolism markers in SHPT patients. There was a positive correlation between BAP and TRAP‐5b in SHPT patients (*r* = .467, *p* = .007), and there was also a positive correlation between PINP and CTX (*r* = .422, *p* = .005). BAP, bone alkaline phosphatase; CTX‐I, type I collagen C‐terminal peptide; PINP, type I collagen amino‐terminal propeptide; SHPT, secondary hyperparathyroidism; TRAP, tartrate‐resistant acid phosphatase.

Correlation analysis of preoperative bone metabolism markers in SHPT patients: There was a positive correlation between BAP and TRAP‐5b in SHPT patients (*r* = .467, *p* = .007), and there was also a positive correlation between PINP and CTX (*r* = .422, *p* = .005). As shown in Figure 1.

### 3.2 Perioperative results

#### 3.2.1 Comparison of mineral bone metabolism‐related indexes in SHPT patients during perioperative period

After PTX, serum phosphorus, serum calcium, iPTH, FGF‐23, CTX‐I, and TRAP‐5b were lower than those before PTX (*p* < .001). Serum BAP level was significantly higher than that before surgery (*p* = .013), serum PINP had an increasing trend compared with that before surgery, and the difference was not statistically significant (*p* = .377) as shown in Table 3.

**Table 3:** Comparison of mineral bone metabolism related indexes in SHPT patients during perioperative period.

	Preoperative	Postoperation	*z/t* value	*p* Value
Ca, mmol/L	2.40 ± 0.27	2.12 ± 0.36	4.570	<.001
P, mmol/L	2.29 ± 0.59	1.03 ± 0.47	13.990	<.001
iPTH, pg/mL	2503.80 (1925.10, 3088.00)	7.00 (1.28, 43.18)	−5.303	<.001
FGF‐23, RU/mL	8756.40 (5390.00, 30226.29)	5123.85 (3567.05, 10619.93)	−3.829	<.001
PINP, μg/L	4707.91 ± 2448.35	5097.06 ± 2983.66	−0.903	.377
BAP, μg/L	194.85 (88.65, 315.72)	640.43 (263.10, 796.99)	−2.490	.013
CTX‐I, μg/L	1.32 (1.14, 1.58)	0.27 (0.20, 0.35)	−4.167	<.001
TRAP‐5b, U/L	15.15 (6.54, 24.05)	6.76 (3.55, 11.81)	−4.107	<.001

*Note*: Values denote mean ± standard deviation, median (interquartile interval). After PTX, serum phosphorus, serum calcium, iPTH, FGF‐23, CTX‐I, and TRAP‐5b were lower than those before PTX (*p* < .001).

Abbreviations: ALB, serum albumin; ALP, alkaline phosphatase; BAP, bone alkaline phosphatase; Ca, serum calcium; CHOL, total cholesterol; CTX‐I, type I collagen C‐terminal peptide; EPO, erythropoietin, FERRITIN; Hb, hemoglobin; P, serum phosphorus; PINP, type I collagen amino‐terminal propeptide; TG, triglyceride; TRAP, tartrate‐resistant acid phosphatase.

#### 3.2.2 Comparison of inflammatory factors in SHPT patients during perioperative period

**Figure 2:**
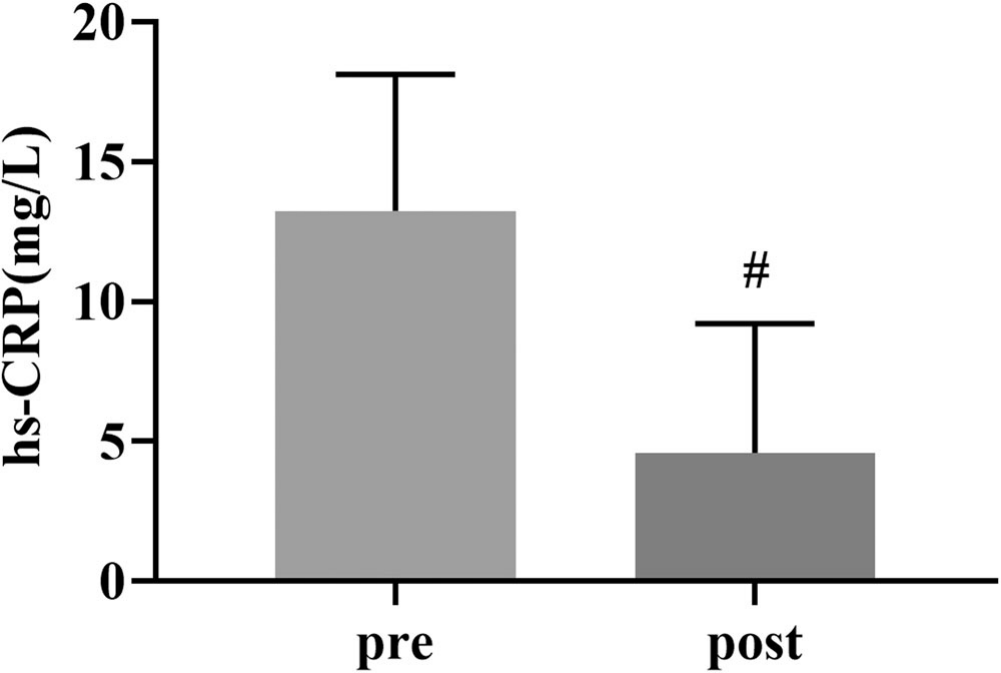
Comparison of inflammatory factors in SHPT patients during perioperative period. The levels of hs‐CRP decreased significantly after operation, and hs‐CRP decreased from 13.14 (9.71, 15.62) (mg/L) before operation to 2.67 (1.73, 5.67) (mg/L) after operation, *p* < .001. SHPT, SHPT, secondary hyperparathyroidism.

The levels of hs‐CRP decreased significantly after operation, and hs‐CRP decreased from 13.14 (9.71, 15.62) (mg/L) before operation to 2.67 (1.73, 5.67) (mg/L) after operation, *p* < .001, as shown in Figure 2.

### 3.3 Cell experiment results

#### 3.3.1 PTHrP in vitro stimulated the expression of inflammatory factor hs‐CRP

**Figure 3:**
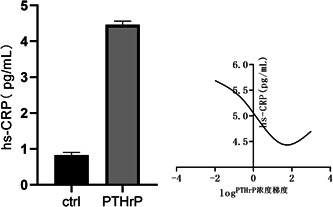
Changes in the expression of inflammatory cytokines hs‐CRP in supernatant in PTHrP stimulation group in LO2. The concentration of hs‐CRP increased after the addition of PTHrP stimulus(1 ng/mL) compared with the blank control group. The expression levels of hs‐CRP in the supernatant of the PTHrP stimulation group (10^−2^, 10^−1^, 1 , 10, and 100 ng/mL) firstly decreased and then increased (*p* < .05). PTHrP, parathyroid hormone‐associated protein.

**Figure 4:**
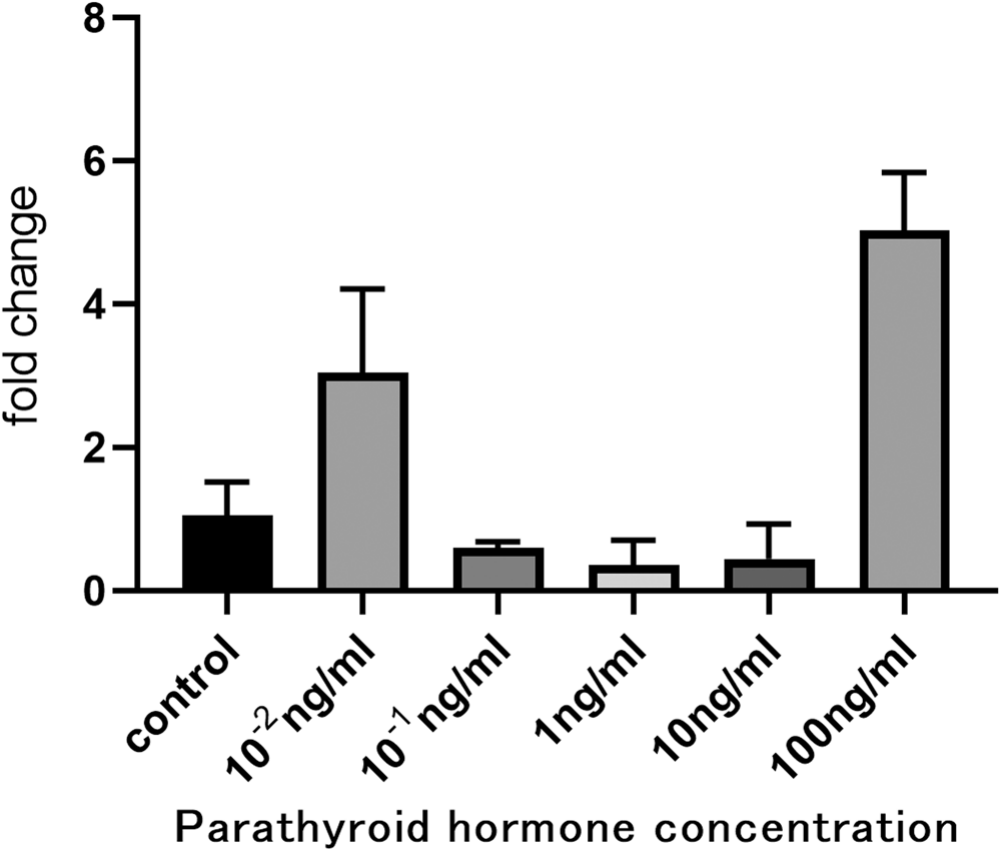
RT‐PCR analysis of hs‐CRP expression in LO2 pretreated with different concentrations of PTHrP. The horizontal coordinate is PTHrP concentration and the vertical coordinate is hs‐CRP expression level. With the increase of PTHrP concentrations level, the level of inflammatory factor hs‐CRP firstly decreased and then increased. When PTHrP concentration was 1 ng/mL, hs‐CRP expression level was the lowest. PTHrP, parathyroid hormone‐associated protein; RT‐PCR, reverse‐transcription polymerase chain reaction.

**Figure 5:**
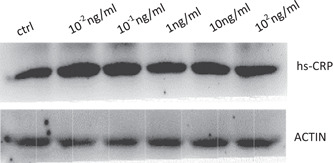
Western blot analysis of hs‐CRP expression in LO2 from Increasing concentrations of PTHrP. With the increase of PTHrP concentrations level, the level of hs‐CRP firstly decreased and then increased. PTHrP, parathyroid hormone‐associated protein.

The expression levels of inflammatory factor hs‐CRP in the supernatant of the PTHrP stimulation group were firstly decreased and then increased, and the inflection point of the level of hs‐CRP in the supernatant appeared when the PTHrP stimulation concentration was about 1 ng/mL, as shown in Figure 3. RT‐PCR and western blot shows the same trend, refer to Figures 4 and 5 for details.

## 4 DISCUSSION

Lancet published the stream survey data on CKD, which showed that as of 2017, CKD patients in the world were nearly 700 million, accounting for 9.1% of the global population, and China had 132.3 million patients, ranking first in the world.^23^ CKD‐MBD is a common complication of CKD, which appears at the early stage of CKD, mainly manifested as calcium and phosphorus metabolism disorder, metastatic calcification, and SHPT.^4^, ^24^ SHPT can lead to calcification of extraceletal vessels and valves, and changes in bone metabolism leading to renal osteodystrophy, increasing the risk of cardiovascular morbidity and mortality.^25^ In this study, 47 SHPT patients were observed to have a statistically significant difference in body mass index compared with the normal control group, and the hemoglobin level was significantly lower than the normal group. In this study, 47 SHPT patients with the dialysis age of (7.11 ± 2.84) years were observed, and the serum iPTH level was significantly higher than the normal range. This indicates that long‐term dialysis patients with CKD are prone to anemia and secondary hyperparathyroidism.

Changes in bone metabolism in patients with SHPT can lead to renal osteodystrophy. Bone biopsy is the gold standard for diagnosis and evaluation of renal osteodystrophy, but it has not been widely used in clinical practice.^26^ In recent years, biomarkers of bone metabolism, which are used as a noninvasive method to evaluate bone metabolism, have emerged. In this study, it was observed that serum levels of bone formation markers BAP and PINP and bone resorption markers CTX‐I and TRAP‐5b in 47 SHPT patients were significantly increased before surgery compared with normal controls, indicating that osteoblasts and osteoclasts had higher activity in SHPT patients.

The study showed that iPTH was positively correlated with PINP, ALP, BAP, and CTX‐I, and that BAP was positively correlated with TRAP‐5b, and PINP was positively correlated with CTX, indicating that iPTH promoted bone formation and also participated in bone resorption, and the activities of osteoclasts and osteoblasts were simultaneously increased in SHPT patients. A domestic study found that there was a positive correlation between iPTH and ALP in SHPT patients, as well as between ALP and TRAP‐5b, and the mechanism of osteoblast‐osteoclast coupling regulation in refractory SHPT patients with high transport bone disease was basically consistent with the results of this study.^24^ Therefore, the obvious abnormality of bone turnover in patients with SHPT may explain the clinical manifestations of bone pain and metastatic calcification.

Microinflammatory state always accompanies CKD and is involved in the process of all‐cause death in patients with CKD.^24^ SHPT patients also had a continuous state of microinflammation, and the level of serum inflammatory factors was increased. We compared the levels of inflammatory cytokines in SHPT patients with normal controls. The results showed that the preoperative serum levels of inflammatory factors hs‐CRP in SHPT patients were significantly higher than those in the healthy control group, indicating to a certain extent that the body inflammation level in patients with ESRD and SHPT was increased. This is consistent with previous studies. CKD progresses to ESRD due to increased chronic inflammation and oxidative stress, with a number of complications such as malnutrition, calcium phosphate abnormalities, atherosclerosis, anemia, and cardiovascular disease.^27^ Long‐term exposure to high levels of hs‐CRP may increase the risk of developing CKD, a large‐scale prospective cohort study suggests.^28^ A recent study showed that hs‐CRP levels were positively associated with the development of diabetic nephropathy.^29^ Previous studies have shown that CRP can be involved in inflammation and kidney damage. For example, CRP can cause kidney damage through the DPP4/CD32b/NF‐kB pathway^30^; CRP promotes renal fibrosis and inflammation through a TGF‐β/SMad3‐dependent mechanism.^31^ In our study, the serum hs‐CRP level was significantly increased in CKD patients with SHPT. We believe that there is inflammation in the body of patients with CKD and SHPT. It is speculated that the development of end‐stage CKD is related to the level of hs‐CRP, and CKD complicated with SHPT may be associated with high level of hs‐CRP. Therefore, we speculate that controlling the level of CRP in patients with CKD may help to delay the progression of CKD and slow down the development of SHPT, but further studies are needed. In conclusion, the inflammatory state of CKD promotes the occurrence of SHPT, and SHPT will further aggravate the inflammation level of patients. The two are harmful positive feedbacks to each other. Improving the inflammatory state of the body may slow down the occurrence of complications of CKD and improve the quality of life of patients.

At present, Few studies have been conducted on mineral bone metabolism‐related indexes and inflammatory factors in kidney diseases, and the results are inconsistent.^20^, ^21^, ^32^, ^33^ The indexes related to mineral bone metabolism and inflammatory factors were significantly changed in SHPT patients. Markers involved in the regulation of bone metabolism included PTH, FGF‐23, calcitonin, vitamin D3, and so forth. We conducted correlation analysis on the indexes related to mineral bone metabolism and inflammatory factors. The results show that inflammatory factors hs‐CRP was significantly positively correlated with iPTH and FGF‐23 levels. We speculate that inflammation may be related to bone metabolism, and controlling inflammation in the body may help to improve the disorder of bone metabolism.

Clinically, for patients with SHPT, it is difficult to be controlled by drugs, surgical excision of the parathyroid gland (PTX) is usually adopted, which is a highly effective treatment method, which can improve the clinical symptoms of patients and reduce the risk of cardiovascular and all‐cause mortality in SHPT patients.^34^ The results of this study showed that, compared with preoperative, the postoperative levels of BAP and PINP in SHPT patients were significantly increased, and the postoperative levels of CTX and TRAP‐5b were significantly decreased, indicating that PTX can promote bone formation and reduce bone resorption, which can partially explain the obvious reduction of postoperative bone pain symptoms in patients. Schneider's team reported that BAP significantly increased in SHPT patients on the 4th day after surgery, while TRAP‐5b rapidly decreased in a short period, which was consistent with our study results.^35^ A longitudinal study involving 31 patients with SHPT found that BAP significantly increased 1 week after PTX, but serum BAP and PINP levels gradually decreased 3 months later.^36^ The Pires team also found a significant decrease in serum BAP in SHPT patients 1 year after PTX,^37^ suggesting that the effect of PTX on bone transport status in SHPT patients may change with the passage of postoperative time. PTX has an opposite effect on bone formation and bone resorption activity, which also proves that PTX does have some influence on SHPT, but the specific signaling pathway and regulatory mechanism are still unclear. In this study, it was found that the levels of serum calcium and serum phosphorus decreased significantly during the perioperative period, as did the levels of FGF‐23. We believe that on the one hand, the decrease of serum phosphorus leads to the decrease of FGF‐23 secretion, on the other hand, the serum iPTH level was independently correlated with FGF‐23, and the iPTH level significantly decreased after PTX, with the loss of the malignant regulation of iPTH and the decrease of FGF‐23 level. It has been reported that PTH can increase the transcription and expression of FGF‐23.^38^ That is, the PTH level of SHPT patients significantly decreased after PTX, leading to the decrease of FGF‐23 level, which is consistent with the present study. We also observed that the levels of inflammatory factors hs‐CRP decreased significantly during the perioperative period, with statistically significant differences, suggesting that PTX can improve the inflammatory state of SHPT patients. In conclusion, successful PTX can reduce bone resorption, improve bone metabolism, and improve bone pain and fracture in patients. In addition, PTX can also reduce the inflammatory state of patients, and reduce the influence of inflammation on complications in patients with CKD.

To further explore the relationship between inflammatory factors and mineral bone metabolism‐related indicators iPTH, we conducted in vitro experiments for further verification. Studies have shown that there are receptors for PTH on the surface of liver cells. CRP is produced by liver cells.^39^ In this study, human liver cells LO2 were selected and treated with different concentrations of PTHrP and constant volume culture medium (blank control) for 24 h. The results showed that with the increase of PTHrP level, the level of inflammatory factor hs‐CRP in the supernatant first decreased and then increased, the same trend was seen at the level of mRNA and protein in cells. In other words, too high or too low PTHrP level can promote inflammation, and there was an optimal PTHrP concentration and the least inflammatory response. We observed a positive correlation between iPTH and inflammatory cytokines in SHPT patients, considering that iPTH levels in all the subjects were at a high level and relatively concentrated. In hemodialysis patients, mortality and incidence of nonfatal cardiovascular events were higher in low parathyroid hormone levels (iPTH < 60 pg/mL) compared to the iPTH ≥600 pg/mL group.^40^ A single‐center retrospective cohort study in China showed that PTH <100 and >300 pg/mL were associated with increased all‐cause mortality than that of PTH 100–200 pg/mL in Chinese PD patients. PTH 100–300 pg/mL might be the best target for Chinese PD patients.^41^ In conclusion, the lower PTH level is not more favorable for HD and PD patients, the presence of PTH in HD and PD patients is within a certain range and the survival time is probably the longest. However, the pathophysiology of clinical CKD patients is significantly changed, and the internal environment is in an extremely disordered state. Whether there is the regulation of inflammatory factors in the most suitable range of PTH concentration, which makes the inflammatory state of the body the lowest. It is still necessary to further expand the sample size to group CKD patients according to PTH concentration, to clarify the changing trend of the influence of PTH on inflammatory factors. Therefore, for patients with refractory hyperparathyroidism, PTH level should not be too low when surgical resection is performed clinically.

There is inflammation in CKD and SHPT patients, and abnormal bone metabolism in SHPT patients. PTH is related to bone formation and bone resorption, and high concentration of PTH is positively correlated with hs‐CRP. Cell experiments suggest that there may be an optimal concentration of PTH to minimize inflammation. Therefore, we speculate that the inflammation and bone metabolism of SHPT patients can be improved by controlling serum PTH level, and controlling inflammation can also delay the progression of CKD and SHPT. For CKD patients with obvious bone pain, nonsteroidal anti‐inflammatory drugs may be used to control the body's inflammation.

The study still has some limitations. The sample size still needs to be expanded. And serum hs‐CRP level needs to be detected at low concentration of PTH in patients with SHPT. Due to poor compliance of patients, follow‐up of enrolled patients was difficult to be advanced, and the corresponding specimens were not retained to re‐detect the levels of bone metabolism‐related indicators and inflammatory factors and conduct statistical analysis. Subsequent experiments such as liver cell PTH receptor antagonist are still needed in the in vitro experiment.

## 5 CONCLUSIONS

In summary, the production of inflammatory factor CRP in SHPT patients is related to mineral bone metabolism indicators. PTH is involved in bone formation and bone resorption, and PTX can significantly improve bone resorption and inflammation state in SHPT patients. In addition, too high or too low PTH level can lead to increased inflammation level, we speculate that there may be an optimal range of PTH concentration to minimize inflammation level in the body. In the future, we still need to further study the specific regulation mechanism of PTH on inflammation, so as to provide theoretical basis for reducing patients' complications and improving survival rate.

## AUTHOR CONTRIBUTIONS

All authors have read and approved the submission of the manuscript.

**Lei Yan**: Conceptualization (lead); writing—original draft (lead); formal analysis (lead); writing—review and editing (equal), funding acquisition (equal). **Qiuyue Xiong**: Writing—original draft (supporting), investigation (equal). **Qin Xu**: Writing—original draft (supporting). **Pengru Ren**: investigation (equal). **Tiantian Li**: investigation (equal). **Huixia Cao**: Methodology (lead); writing—review and editing (equal); formal analysis (supporting); funding acquisition (equal). **Fengmin Shao**: Conceptualization (supporting), methodology (supporting), writing—review and editing (equal), project administration (lead).

## ETHICS STATEMENT

All patients signed the written informed consent. All procedures were approved by Medical Ethics Committee of Henan Provincial People's Hospital (No. 2020.206).
